# A nomogram model integrating radiomics and clinical variables to predict napsin a expression in lung adenocarcinoma patients

**DOI:** 10.3389/fonc.2025.1595406

**Published:** 2025-07-04

**Authors:** Bo Pang, LiNa Liu, Man Gao, ChongHai Xu, Zhaisong Gao, ZhiChao Wang, JianZhong Guan

**Affiliations:** ^1^ Nuclear Medicine Department, 971 Hospital, People's Liberation Army Navy, Qingdao, Shandong, China; ^2^ Orthopaedic Department, 971 Hospital, People's Liberation Army (PLA) Navy, Qingdao, Shandong, China

**Keywords:** lung adenocarcinoma, napsin a, nomogram, radiomics, non-invasive diagnosis

## Abstract

**Background:**

Lung adenocarcinoma, a major subtype of non-small cell lung cancer, requires non-invasive diagnostic tools to improve early detection and differentiate primary from metastatic tumors. Napsin A, a key marker for primary lung adenocarcinoma, is traditionally assessed via invasive biopsy, limiting its utility in reflecting tumor heterogeneity. Radiomics, which extracts quantitative features from medical images, offers potential for non-invasive prediction of molecular markers like Napsin A.

**Objectives:**

To develop and validate a nomogram integrating radiomic features and clinical variables for non-invasive prediction of Napsin A expression in lung adenocarcinoma.

**Methods:**

This retrospective study enrolled 308 lung adenocarcinoma patients (training cohort: n = 246; validation cohort: n = 62), with contrast-enhanced CT images were used to extract 1,734 radiomic features, which underwent dimensionality reduction via t-tests, Pearson correlation, minimum redundancy maximum relevance (mRMR), and LASSO regression, retaining 27 final features; significant clinical variables (gender, smoking history, pulmonary cavity, spiculation sign, pleural indentation sign) were selected by logistic regression. A nomogram integrating radiomic and clinical predictors was developed and evaluated using ROC curves (AUC for Napsin A prediction), calibration curves (Hosmer-Lemeshow test), and decision curve analysis (DCA) for clinical utility.

**Results:**

The integrated nomogram model outperformed standalone radiomic and clinical models in predicting Napsin A expression, achieving AUC values of 0.844 (95% CI: 0.790–0.898) in the training cohort (n = 246) and 0.845 (95% CI: 0.724–0.967) in the validation cohort (n = 62), with balanced accuracy of 82.1% and 80.6%, respectively. Calibration curves showed strong agreement between predicted and observed outcomes (Hosmer-Lemeshow P > 0.05), and decision curve analysis confirmed its superior clinical utility across diverse threshold probabilities.

**Conclusion:**

The integrated nomogram offers a reliable non-invasive method for predicting Napsin A expression in lung adenocarcinoma, supporting personalized treatment decisions and reducing reliance on invasive biopsies.

## Introduction

Lung adenocarcinoma typically presents with subtle early symptoms, leading to late-stage diagnoses in most patients. Current data indicate that the overall 5-year survival rate for lung adenocarcinoma is approximately 19% globally. For advanced-stage patients, this rate is lower than 20%, underscoring the critical need for improved early detection methods (1–3). Given that delayed diagnosis of lung adenocarcinoma often results in advanced-stage disease, which is associated with limited therapeutic options, higher metastatic potential, and dismal survival outcomes, early identification of the disease is pivotal for enabling curative interventions and improving patient survival (4, 5).

Napsin A serves as a crucial diagnostic marker for distinguishing primary lung adenocarcinoma from metastatic lung cancer, with its expression levels also linked to tumor differentiation. Current studies have demonstrated that immunohistochemical detection of Napsin A expression aids in the diagnosis and differentiation of lung adenocarcinoma (6–8). However, traditional tissue biopsy and immunohistochemical detection have certain limitations. Tissue biopsy is an invasive procedure associated with significant risks, including pneumothorax (23.2–27% incidence in CT-guided lung biopsies, with 6–10% requiring chest tube insertion), clinically significant bleeding (7.9% in transbronchial cryobiopsies, linked to traction bronchiectasis and large vessel involvement), and infection (<1% in CT-guided procedures). Key risk factors for pneumothorax include small lesion size (≤2 cm), needle traversal of pulmonary fissures, and emphysema, while bleeding risk increases with traction bronchiectasis on imaging and histologic presence of medium-large vessels (9). Additionally, immunohistochemistry can only assess a small portion of tumor tissue, which may not fully capture tumor heterogeneity, potentially affecting detection accuracy (10, 11). Therefore, exploring a non-invasive diagnostic method that can comprehensively reflect tumor heterogeneity is an urgent need.

Radiomics enables non-invasive characterization of tumor biology by translating medical images into quantitative features that correlate with histopathological and molecular traits. For instance, radiomic texture features derived from CT images, such as those from gray-level co-occurrence matrices (GLCM), have been shown to reflect tumor cellularity, stromal fibrosis, and vascular density, which are directly linked to adenocarcinoma differentiation and Napsin A expression. Recent studies further indicate that shape features, such as spiculation or margin irregularity, may correlate with invasive growth patterns and reduced marker expression by capturing desmoplastic reactions in the tumor microenvironment (12–14).

In recent years, radiomics has emerged as a promising research area that has made remarkable progress in the diagnosis, pathological staging, and gene mutation prediction of lung adenocarcinoma, offering new approaches for precise tumor diagnosis and treatment (15, 16). Notably, no prior studies have integrated radiomic features with clinical variables to develop predictive models for Napsin A expression. This study therefore represents an early attempt to bridge radiomics and clinical data for non-invasive assessment of this critical molecular marker. Napsin A expression levels are linked to lung adenocarcinoma differentiation, potentially impacting treatment decisions and patient outcomes. Consequently, creating a predictive model using radiomic features and clinical variables is crucial for personalized lung adenocarcinoma treatment.

This study aimed to extract radiomic features associated with Napsin A expression from contrast-enhanced CT images and construct a nomogram model incorporating clinical variables to provide a novel non-invasive prediction tool for lung adenocarcinoma diagnosis and treatment.

## Materials and methods

### Patients

Clinical data for 308 lung adenocarcinoma patients were retrospectively collected from the Hospital Information System (HIS) and pathological archives of the 971st Hospital of the PLA Navy between January 2018 and July 2022. Data included age, sex, smoking history, and TNM staging (AJCC 8th edition), all of which were extracted from electronic health records (EHRs). Smoking status was documented based on patient self-reporting in clinical notes and verified through medical history entries. Radiographic parameters (e.g., pulmonary cavity, spiculation sign) were assessed by two senior radiologists (≥8 years of chest imaging experience) via consensus review of contrast-enhanced CT images, with discrepancies resolved through discussion to ensure inter-observer consistency. All data were de-identified to protect patient privacy. The study was approved by the Ethics Committee of 971 Hospital, PLA Navy (Ethics Approval No. 20250321), and informed consent was waived due to its retrospective nature.

### Inclusion criteria

1. Histopathologically confirmed lung adenocarcinoma according to the 2021 WHO Classification of Lung Tumors, verified by two senior pathologists (≥10 years of experience) via hematoxylin-eosin (H&E) staining (17).
2. Underwent contrast-enhanced chest CT within 1 month prior to biopsy or surgery, with clear visualization of the primary tumor.
3. Available immunohistochemical staining results for Napsin A using anti-Napsin A monoclonal antibody (Clone IP64, Leica Biosystems) with a positive control.
4. Complete clinical data, including age, sex, smoking history (defined as ≥100 cigarettes lifetime), and AJCC 8th edition TNM staging.

### Exclusion criteria

**Figure 1:**
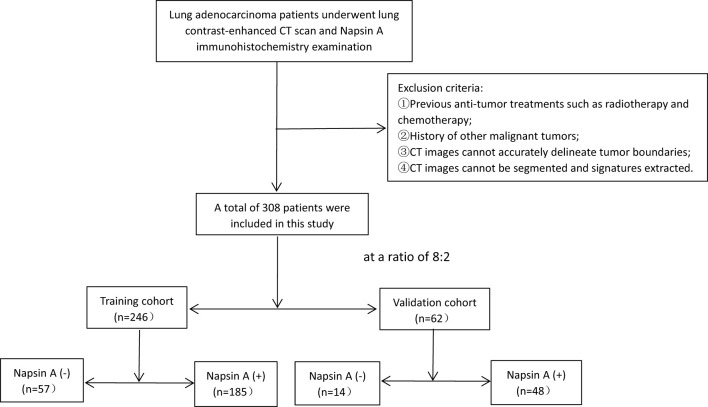
Flowchart of patient selection. Exclusion criteria and corresponding excluded patient numbers are clearly labeled [① prior anti-tumor treatment, (n=12); ② history of other malignancies, (n=15); ③ unclear tumor boundaries,(n=21); ④ unsegmentable CT images, (n=18)], ensuring transparency in cohort construction.

1. Prior neoadjuvant chemotherapy, radiotherapy, or targeted therapy before CT scanning or biopsy.
2. Active diagnosis of other primary malignancies (except non-melanoma skin cancer).
3. Unclear tumor boundaries defined as the inability to delineate tumor margins from adjacent atelectasis or inflammation via multi-planar reconstruction (MPR) by consensus of two radiologists (≥8 years of experience) through qualitative visual assessment; poor image quality including motion artifact score ≥3 on a 5-point scale (1 = minimal artifact, 5 = non-diagnostic), characterized by blurring or misregistration of anatomical structures compromising tumor contouring, or arterial phase contrast enhancement <20 Hounsfield Units (HU) (measured as the mean attenuation difference between tumor and adjacent normal lung tissue), indicating inadequate vascular enhancement for reliable radiomic feature extraction.
4. Pathological inadequacy: Biopsy specimens with <10 viable tumor cells or core biopsy length <10 mm. Failed immunohistochemistry staining (e.g., nonspecific background staining or technical errors).
5. Severe comorbidities (e.g., decompensated heart failure, active pulmonary infection) precluding curative-intent treatment.
  Patients were randomized into training (n = 246) and validation (n = 62) cohorts at an 8:2 ratio using a random number table (**Figure 1**), performed by an independent statistician and blinded to radiologists and pathologists. Baseline characteristics (age, sex, smoking history, tumor stage) and radiological features (cavity sign, lobulation sign, etc.) were balanced between cohorts (P>0.05, **Tables 1**, **2**).

**Table 1:** Clinical parameters of patients with napsin A expression in training and validation cohorts.

Clinical parameters	Data	Training cohort (n = 246)			Validation cohort (n = 62)			^2^ value	*P* value
		Napsin A (-)	Napsin A (+)	*P* value	Napsin A (-)	Napsin A (+)	*P* value		
Age	62.63 ± 9.61	64.84 ± 7.52	62.26 ± 9.90	0.070	62.07 ± 10.99	61.75 ± 10.17	0.919		0.449
Gender				**<0.001**			0.251	3.135	0.077
Male	165	42 (73.68%)	96 (49.21%)		8 (57.14%)	19 (39.58%)			
Female	143	15 (26.32%)	93 (50.79%)		6 (42.86%)	29 (60.42%)			
Smoking				**0.001**			**0.031**	0.548	0.459
Yes	151	38 (66.67%)	80 (42.33%)		11 (78.57%)	22 (45.83%)			
No	157	19 (33.33%)	109 (57.67%)		3 (21.43%)	26 (54.17%)			
Primary tumor staging				0.603			0.182	1.521	0.677
T1	63	9 (15.79%)	42 (22.22%)		3 (21.43%)	9 (18.75%)			
T2	103	21 (36.84%)	61 (32.28%)		7 (50.00%)	14 (29.17%)			
T3	68	14 (24.56%)	43 (22.75%)		2 (14.29%)	9 (18.75%)			
T4	74	13 (22.81%)	43 (22.75%)		2 (14.29%)	16 (33.33%)			
Lymph node metastasis staging				0.540			0.814	0.393	0.942
N0	138	25 (43.86%)	85 (44.97%)		7 (50.00%)	21 (43.75%)			
N1	109	19 (33.33%)	69 (36.51%)		4 (28.57%)	17 (35.42%)			
N2	60	12 (21.05%)	35 (18.52%)		3 (21.43%)	10 (20.83%)			
N3	1	1 (1.75%)	0						

Age was shown as mean ± standard deviation, and other data were the number of patients with the percentage in parentheses. The P value marked bold indicated statistical significance.

Napsin A (-) indicated negative expression of Napsin A, while Napsin A (+) indicated positive expression of Napsin A.

**Table 2:** Radiography parameters of patients with the expression of Napsin A in the training cohort and validation cohort.

Radiography parameters	Data	Training cohort (n = 246)			Validation cohort (n = 62)			^2^ value	*P* value
		Napsin A (-)	Napsin A (+)	*P* value	Napsin A (-)	Napsin A (+)	*P* value		
Pulmonary cavity				**0.016**			**<0.001**	2.368	0.124
No	279	48 (84.21%)	178 (94.18%)		8 (57.14%)	45 (93.75%)			
Yes	29	9 (15.79%)	11 (5.82%)		6 (42.86%)	3 (6.25%)			
Vacuolar sign				**<0.001**			**<0.001**	3.034	0.082
No	239	35 (61.40%)	161 (85.19%)		4 (28.57%)	39 (81.25%)			
Yes	69	22 (38.60%)	28 (14.81%)		10 (71.43%)	9 (18.75%)			
Lobulation sign				0.945			0.271	0.956	0.328
No	13	2 (3.51%)	7 (3.70%)		0	4 (8.33%)			
Yes	195	55 (96.49%)	182 (96.30%)		14 (100%)	44 (91.67%)			
Spiculation sign				0.529			0.169	0.549	0.459
No	23	5 (8.77%)	12 (6.35%)		0	6 (12.50%)			
Yes	285	52 (91.23%)	177 (93.65%)		14 (100%)	42 (87.50%)			
Pleural indentation sign				**0.023**			0.510	0.127	0.722
No	128	16 (28.07%)	85 (44.97%)		5 (35.71%)	22 (45.83%)			
Yes	180	41 (71.93%)	104 (55.03%)		9 (64.29%)	26 (54.17%)			
Air bronchogram sign				0.840			0.721	1.871	0.171
No	261	48 (84.21%)	157 (83.07%)		13 (92.86%)	43 (89.58%)			
Yes	47	9 (15.79%)	32 (16.93%)		1 (7.14%)	5 (10.42%)			
Vascular convergence sign				0.412			0.150	0.365	0.546
No	114	18 (31.58%)	71 (37.57%)		8 (57.14%)	17 (35.42%)			
Yes	194	39 (68.42%)	118 (62.43%)		6 (42.86%)	31 (64.58%)			

All data were the number of patients with the percentage in parentheses. The P value marked bold indicated statistical significance.

Napsin A (-) indicated negative expression of Napsin A, while Napsin A (+) indicated positive expression of Napsin A.

### CT scanning

All patients received contrast-enhanced CT scans with an Aquilion ONE 640 CT scanner (Toshiba, Tokyo, Japan).Patients were positioned supine with both hands raised above their heads.Scanning ranged from the apex of the lungs to the top of the adrenal glands after breath-holding at the end of inhalation.

All contrast-enhanced CT scans were acquired using a Toshiba Aquilion ONE 640-slice CT scanner with a standardized 120 kV tube voltage and automatic tube current modulation (100–400 mAs, adjusted based on patient characteristics such as BMI), 512×512 matrix, 1 mm slice thickness/interval, and pitch 1.375:1. Iodixanol (350 mgI/mL, Visipaque, GE Healthcare) was administered at a fixed dose of 80–100 mL (3.5 mL/s injection rate), consistent with standard clinical protocols for chest CT in lung cancer patients, to ensure uniform contrast enhancement for radiomic feature extraction without weight-based adjustment.

### Immunohistochemical evaluation of napsin A expression

**Figure 2:**
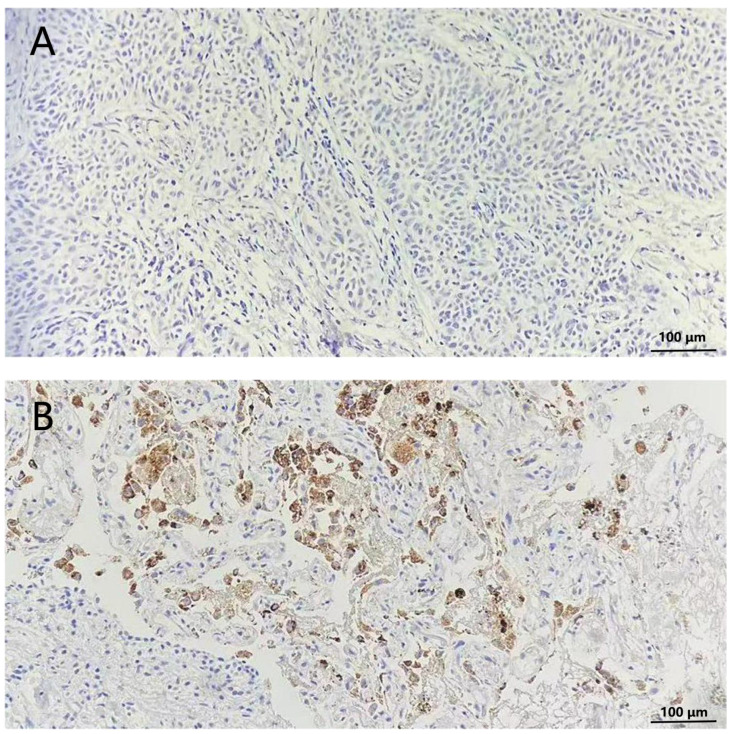
Immunohistochemical staining of Napsin A in lungs of non-small cell lung cancer patients (200×). **(A)** Cells without stained indicated negative expression of Napsin **(A, B)** cytoplasm stained tan indicated positive expression of Napsin A.

For immunohistochemical assessment of Napsin A expression, lung adenocarcinoma tissue specimens were prepared in 4-micron thick slices and immunohistochemically stained with anti-Napsin A antibodies (Clone number: IP64, Leica Biosystems).Two experienced pathologists independently assessed the staining results, remaining blinded to both the clinical data of the patients and the Napsin A expression status. Cells with negative Napsin A expression showed no coloration, while cells with positive Napsin A expression displayed brown-yellow cytoplasm (**Figure 2**).

### Tumor segmentation

CT images were exported from the PACS in DICOM format and underwent standardized preprocessing to ensure radiomic feature reproducibility, as spatial resolution and intensity normalization significantly impact feature stability (18). Preprocessing steps included:

1. Isotropic voxel resampling: Images were resampled to 1×1×1 mm³ isotropic resolution using 3D-Slicer, correcting for original slice thickness variations to standardize spatial sampling across patients.
2. Grayscale normalization: Pixel values were normalized to the range of (–1, 1) to minimize intensity bias between scans.
3. Gaussian filtering (σ=1.0): Applied to reduce noise while preserving structural features, using PyRadiomics for automated processing.

These were automated using 3D-Slicer (Version 4.11; https://www.slicer.org) and PyRadiomics (Version 3.0.1; http://pyradiomics.readthedocs.io).

**Figure 3:**
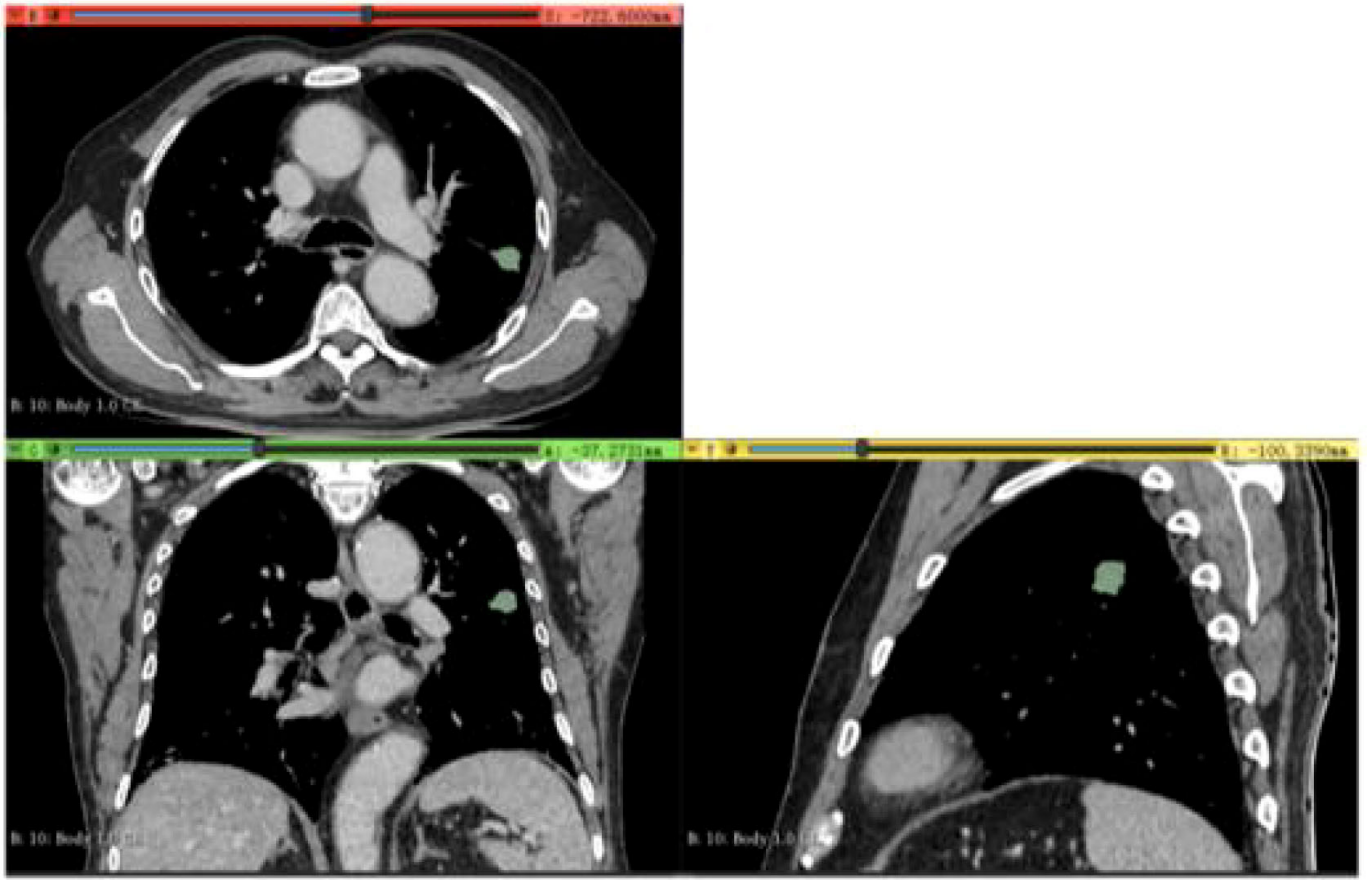
Delineation of Region of Interest (ROI) in a 65-Year-Old Male Patient (Validation Cohort). Delineation of region of interest (ROI) in a 65-year-old male patient (validation cohort) using portal venous phase CT scan exported from 3D Slicer software. Axial, coronal, and sagittal views with three-dimensional ROI reconstruction (green area) are shown.

Two experienced radiologists, each with over five years in chest CT imaging diagnosis, manually segmented tumor regions of interest (ROIs) using 3D-Slicer software (Version 4.11, https://www.slicer.org, USA) (**Figure 3**). To ensure an unbiased assessment, both radiologists were blinded to the patients’ clinical data and Napsin A expression status.

To assess inter-observer and intra-observer reproducibility, two radiologists independently outlined ROIs on 30 randomly chosen CT images, followed by the extraction of radiomic features using PyRadiomics (v3.0.1, http://pyradiomics.readthedocs.io).Agreement was evaluated using intraclass correlation coefficients (ICC).Two weeks later, one radiologist repeated the delineation on the same 30 CT images to assess intra-observer reproducibility. Radiomic features with ICC values exceeding 0.75 in both inter- and intra-observer evaluations were deemed stable and selected for further analysis.

### Radiomic feature extraction

Radiomic features were extracted from the segmented tumors using PyRadiomics (v3.0.1, http://pyradiomics.readthedocs.io), an open-source library adhering to the guidelines of the Image Biomarker Standardization Initiative. Each patient’s venous phase CT images yielded 1,734 radiomic features. These features were classified into intensity, shape, and various matrix types, including grey level co-occurrence, gray level run-length, grey level zone size, and neighborhood gray-tone difference matrices.

#### Feature selection and model development

To balance feature relevance and model simplicity, we employed a hybrid mRMR-LASSO pipeline, prioritizing interpretability and biological plausibility over purely dimensionality-reduction-focused methods like principal component analysis (PCA). Unlike PCA, which transforms features into orthogonal components that may obscure biological interpretability, mRMR retains features with maximal relevance to the outcome (Napsin A expression) and minimal inter-feature redundancy, while LASSO introduces sparsity to identify a parsimonious set of predictors. This approach ensures that selected features have direct statistical and biological links to the target variable, aligning with radiomics best practices for molecular prediction.

To assess the impact of feature selection methods, we conducted a parallel analysis using PCA to reduce features to the same dimensionality (27 features) and compared model performance. The PCA-derived model achieved an AUC of 0.781 (95% CI: 0.723–0.839) in the training cohort and 0.779 (95% CI: 0.658–0.900) in the validation cohort, significantly lower than the mRMR-LASSO model’s AUC of 0.844 and 0.845, respectively (P<0.05, DeLong test). This suggests that the mRMR-LASSO pipeline better preserves feature relevance for Napsin A prediction, likely due to its focus on outcome-driven feature selection rather than global variance maximization. The LASSO model’s optimal λ value was determined using 10-fold cross-validation, focusing on minimizing error. The final model included 27 features, selected based on non-zero coefficients at the optimal λ value of 0.02.

The selected 27 radiomic features were utilized in multiple machine learning algorithms, such as logistic regression, support vector machine, K-Nearest Neighbor, decision tree, random forest, extra trees, extreme gradient boosting, light gradient boosting machine, and multilayer perceptron. The optimal radiomic model was identified through 5-fold cross-validation.

Clinical variables underwent one-way ANOVA, and those with P<0.05 were further examined using multivariate analysis. Significant clinical variables (P<0.05) were selected to construct a clinical model using the optimal algorithm identified in the radiomic model development.

### Nomogram construction and evaluation

A nomogram model was developed by combining radiomic and clinical models. This nomogram visually represents the relationship between individual predictors and the probability of Napsin A expression. The contribution of each variable is displayed as a point scale, allowing for straightforward calculation of the total score and corresponding predicted probability.

The nomogram’s performance was assessed through receiver operating characteristic (ROC) curves, with area under the curve (AUC) values computed for both the training and validation cohorts. Calibration was evaluated using the Hosmer-Lemeshow test to compare predicted probabilities with observed outcomes. Decision curve analysis (DCA) assessed the clinical utility of the nomogram model in comparison to the standalone radiomic and clinical models.

### Statistical analysis

SPSS 25.0 software was used for statistical analyses. Independent sample t-tests were applied to variables with normal distribution, whereas Mann-Whitney U tests were utilized for those without. Categorical data were analyzed using χ² tests. Results are presented as mean ± standard deviation (x ± s), with statistical significance set at P<0.05.

## Results

### Patient characteristics

The study included 308 lung adenocarcinoma patients with a mean age of 63.2 ± 8.9 years and a sex distribution of 53.6% male. Patients were randomized into a training cohort (n = 246) and a validation cohort (n = 62). In the training cohort, Napsin A positivity was observed in 189 patients (76.9%), with females and non-smokers showing significantly higher positivity rates (P<0.05). Key clinical and radiological characteristics were balanced between cohorts (P>0.05), as detailed in **Tables 1**, **2**.

In the training cohort, Napsin A positivity was notably more prevalent in female and non-smoking patients than in their male and smoking counterparts (P<0.05). The radiographic features analyzed in **Table 2** were selected based on their established associations with tumor biology and Napsin A expression in prior studies. For example, pulmonary cavity is rarely observed in pure adenocarcinoma and is more characteristic of squamous cell carcinoma, aligning with our finding that its absence correlated with Napsin A positivity. Spiculation sign and pleural indentation, markers of desmoplastic reactions, have been linked to tumor invasiveness and reduced differentiation in EGFR-mutant adenocarcinomas (19), which may explain their association with Napsin A negativity in our cohort. The vacuolar sign, indicative of lepidic growth, correlates with well-differentiated adenocarcinoma subtypes and higher Napsin A expression (20, 21), consistent with its prevalence in Napsin A-positive cases. These features were prioritized for their potential to reflect tumor microenvironment and differentiation status, which are biologically linked to Napsin A expression as a marker of alveolar epithelial origin.

### Radiomic feature selection

**Figure 4:**
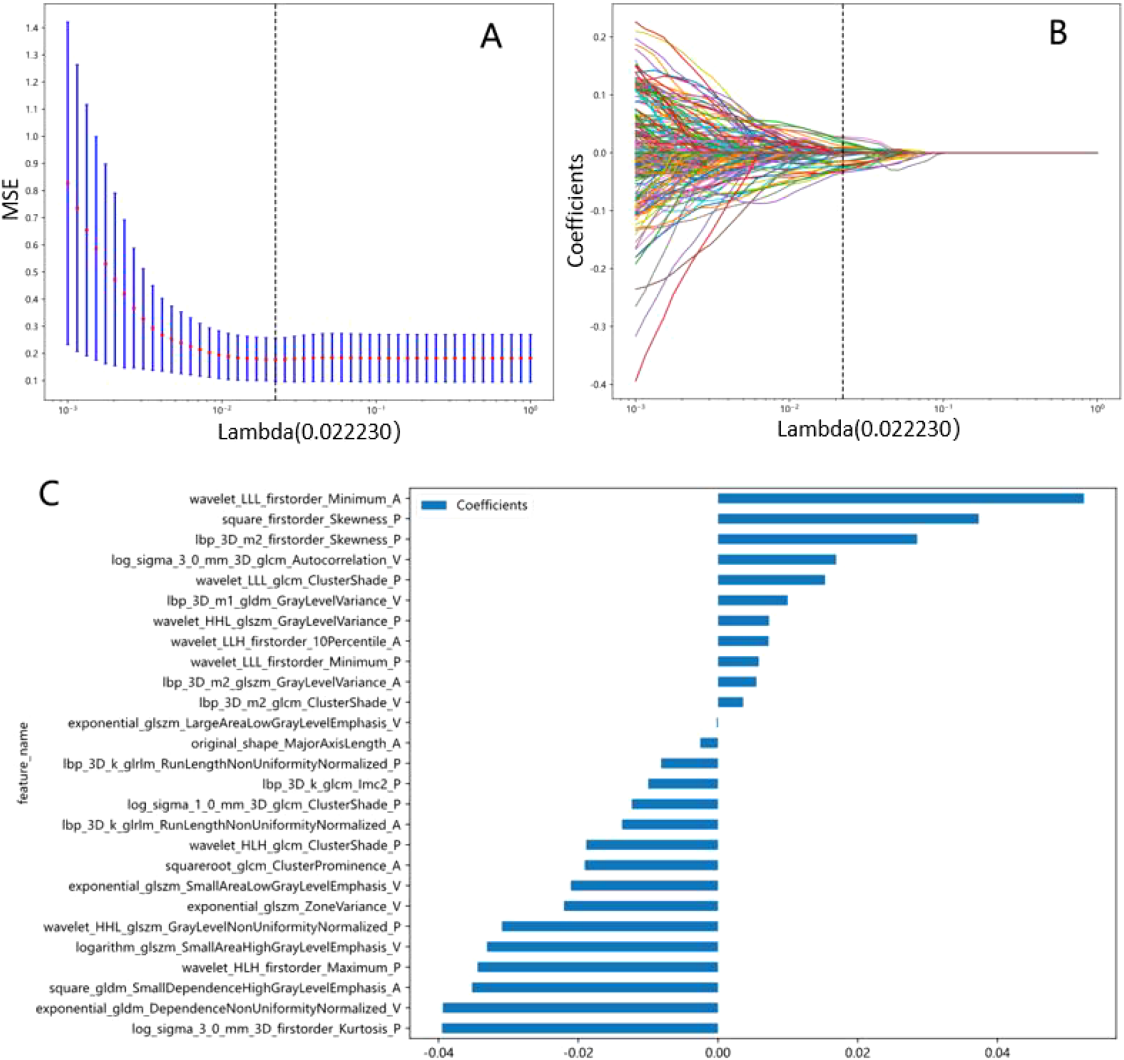
Radiomics signatures associated with Napsin A expression were selected using LASSO regression models. **(A)** Cross-validation curve. An optimal λ value (λ=0.02) was selected by 10-fold cross validation, and 27 non-zero coefficients signatures were chosen. **(B)** LASSO coefficient profiles of the 1734 radiomics signatures against the deviance explained. **(C)** Histogram shows the contribution of the selected signatures with their regression coefficients in the signature construction.

After the multi-step feature selection process, 27 radiomic features with the highest predictive value for Napsin A expression were retained (**Figures 4A, B**).The optimal λ value in the LASSO model was 0.02, and the corresponding coefficients for each feature were calculated (**Figure 4C**).The Rad-score was calculated by summing the selected features, each weighted by its respective coefficient (**Table 3**).

**Table 3:** Image omics feature list.

Feature name	coefficient	Feature type
lbp_3D_k_ghim_RmLengthNonUniformityNormalized_A	-0.013737	GLRLM
lbp_3D_m2_glszm_GrayLevelVariance_A	+0.005512	GLSZM
original_shape_MajorAxisLength_A	-0.002549	Shape
square_gldm_SmallDependenceHighGrayLevelEmphasis_A	-0.035210	GLDM
squarroot_glcm_ClusterProminence_A	-0.019069	GLCM
wavelet_LILI_firstorder_10Percentile_A	+0.007258	Wavelet
wavelet_LILI_firstorder_Mnimrum_A	+0.052376	Wavelet
lbp_3D_k_glcm_Imc2_P	-0.009993	GLCM
lbp_3D_k_ghlm_RmLengthNonUniformityNormalized_P	-0.008154	GLRLM
lbp_3D_m2_firstorder_Skewness_P	+0.028505	First Order
log_sigma_1_0_nm_3D_glcm_ClusterShade_P	-0.012343	GLCM
log_sigma_3_0_nm_3D_firstorder_Kurtosis_P	-0.039497	First Order
square_firstorder_Skewness_P	+0.037324	First Order
wavelet_HHL_glszm_GrayLevelNonUniformityNormalized_P	-0.030927	GLSZM
wavelet_HHL_glszm_GrayLevelVariance_P	+0.007324	GLSZM
wavelet_HLH_firstorder_Maximum_P	-0.034408	First Order
wavelet_HLH_glcm_ClusterShade_P	-0.018865	GLCM
wavelet_LILI_firstorder_Mnimrum_P	+0.005854	Wavelet
wavelet_LILI_glcm_ClusterShade_P	+0.015338	GLCM
exponential_gldm_DependenceNonUniformityNormalized_V	-0.039399	GLDM
exponential_glszm_LargeAreaLowGrayLevelEmphasis_V	-0.000192	GLSZM
exponential_glszm_SmallAreaLowGrayLevelEmphasis_V	-0.021068	GLSZM
exponential_glszm_ZoneVariance_V	-0.022050	GLSZM
lbp_3D_m1_gldm_GrayLevelVariance_V	+0.009984	GLDM
lbp_3D_m2_glcm_ClusterShade_V	+0.003619	GLCM
log_sigma_3_0_nm_3D_glcm_Autocorrelation_V	+0.016929	GLCM
logarithm_glszm_SmallAreaHighGrayLevelEmphasis_V	-0.033070	GLSZM

### Model performance

Logistic regression (LR) demonstrated optimal performance among the tested machine learning algorithms for both radiomic and clinical models. The radiomic model attained an AUC of 0.80 in both the training (95% CI, 0.73-0.87) and validation cohorts (95% CI, 0.68-0.93) as shown in **Table 4**. The clinical model, which includes gender, smoking history, pulmonary cavity, spiculation sign, and pleural indentation sign, demonstrated AUC values of 0.72 (95% CI: 0.65-0.80) in the training cohort and 0.82 (95% CI: 0.68-0.96) in the validation cohort.

**Table 4:** The performance of different machine learning algorithms in prediction of Napsin A expression in lung adenocarcinoma.

Machine learning algorithms	AUC	95% CI	Accuracy (%)	Sensitivity (%)	Specificity (%)	PPV (%)	NPV (%)
Logistic regression							
Training cohort	0.798	0.731 - 0.865	82.9	70.4	75.4	83.0	82.6
Validation cohort	0.804	0.676 - 0.932	82.3	62.5	85.7	82.5	80.0
Support vector machine							
Training cohort	0.899	0.836 - 0.962	85.4	91.0	87.7	84.0	100
Validation cohort	0.741	0.591 - 0.891	77.4	60.4	78.6	77.4	0
K-NearestNeighbor							
Training cohort	0.846	0.799 - 0.894	81.7	85.7	66.7	82.4	75.0
Validation cohort	0.508	0.343 - 0.673	79.0	100	12.5	78.7	100
Decision tree							
Training cohort	1.000	0.999 - 1.000	99.2	98.9	100	99.5	98.2
Validation cohort	0.482	0.355 - 0.610	62.9	100	0	76.6	20.0
Random forest							
Training cohort	0.997	0.993 - 1.000	95.5	98.9	100	95.4	96.0
Validation cohort	0.593	0.434 - 0.752	77.4	22.9	100	78.3	50.0
Extra trees							
Training cohort	1.000	0.999 - 1.000	99.2	98.9	100	99.5	98.2
Validation cohort	0.573	0.407 - 0.739	71.0	68.8	66.7	75.9	0
Extreme gradient boosting							
Training cohort	0.998	0.994 - 1.000	98.8	98.4	100	98.9	98.2
Validation cohort	0.707	0.516 - 0.898	80.6	97.9	50.0	80.0	100
Light gradient boosting machine							
Training cohort	0.970	0.952 - 0.989	78.5	92.6	93.0	78.1	100
Validation cohort	0.693	0.520 - 0.867	77.4	68.8	71.4	77.4	0
Multilayer perceptron							
Training cohort	0.826	0.765 - 0.887	82.1	93.7	54.4	81.4	93.3
Validation cohort	0.789	0.667 - 0.911	80.6	56.3	92.9	80.0	100

AUC, area under the curve; 95%CI, confidence interval; PPV, positive predictive value; NPV, negative predictive value.

### Nomogram model and predictive utility

**Figure 5:**
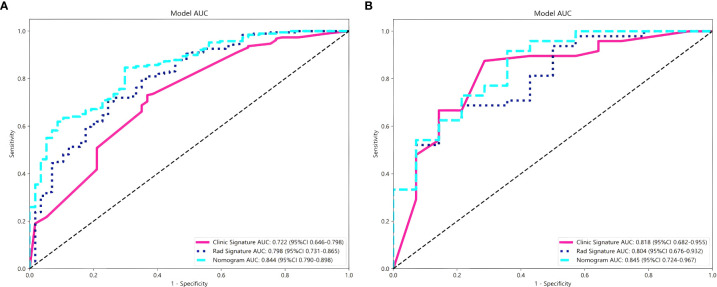
Comparison of the performance of three models for predicting Napsin A expression in lung adenocarcinoma. ROC curves for radiomics signature model, clinical signature model and nomogram model in training cohort **(A)** and validation cohort **(B)**.

The combined nomogram model, incorporating both radiomic features and clinical variables, showed enhanced performance with an AUC of 0.84 (95% CI: 0.79–0.90) in the training cohort (**Figure 5A**) and 0.85 (95% CI: 0.72–0.97) in the validation cohort (**Figure 5B**). **Table 5** indicates that the nomogram model outperformed other models in balanced accuracy, achieving 77.5% in the training cohort and 78.0% in the validation cohort, compared to the radiomic model**’**s 72.9% and 74.1%, and the clinical model**’**s 68.7% and 82.2%, respectively.

**Table 5:** Predictive performance of the three models in training cohort and validation cohort.

Models	AUC	95% CI	Accuracy (%)	Sensitivity (%)	Specificity (%)	PPV (%)	NPV (%)
Radiomics signature model							
Training cohort	0.798	0.731 - 0.865	82.9	70.4	75.4	83.0	82.6
Validation cohort	0.804	0.676 - 0.932	82.3	62.5	85.7	82.5	80.0
Clinical signature model							
Training cohort	0.722	0.646 - 0.798	78.9	73.0	64.3	79.7	66.7
Validation cohort	0.818	0.682 - 0.955	82.3	87.5	76.9	83.6	71.4
Nomogram model							
Training cohort	0.844	0.790 - 0.898	82.1	84.7	70.2	81.9	84.2
Validation cohort	0.845	0.724 - 0.967	80.6	91.7	64.3	80.0	100

AUC, area under the curve; 95%CI, confidence interval; PPV, positive predictive value; NPV, negative predictive value.

**Figure 6:**
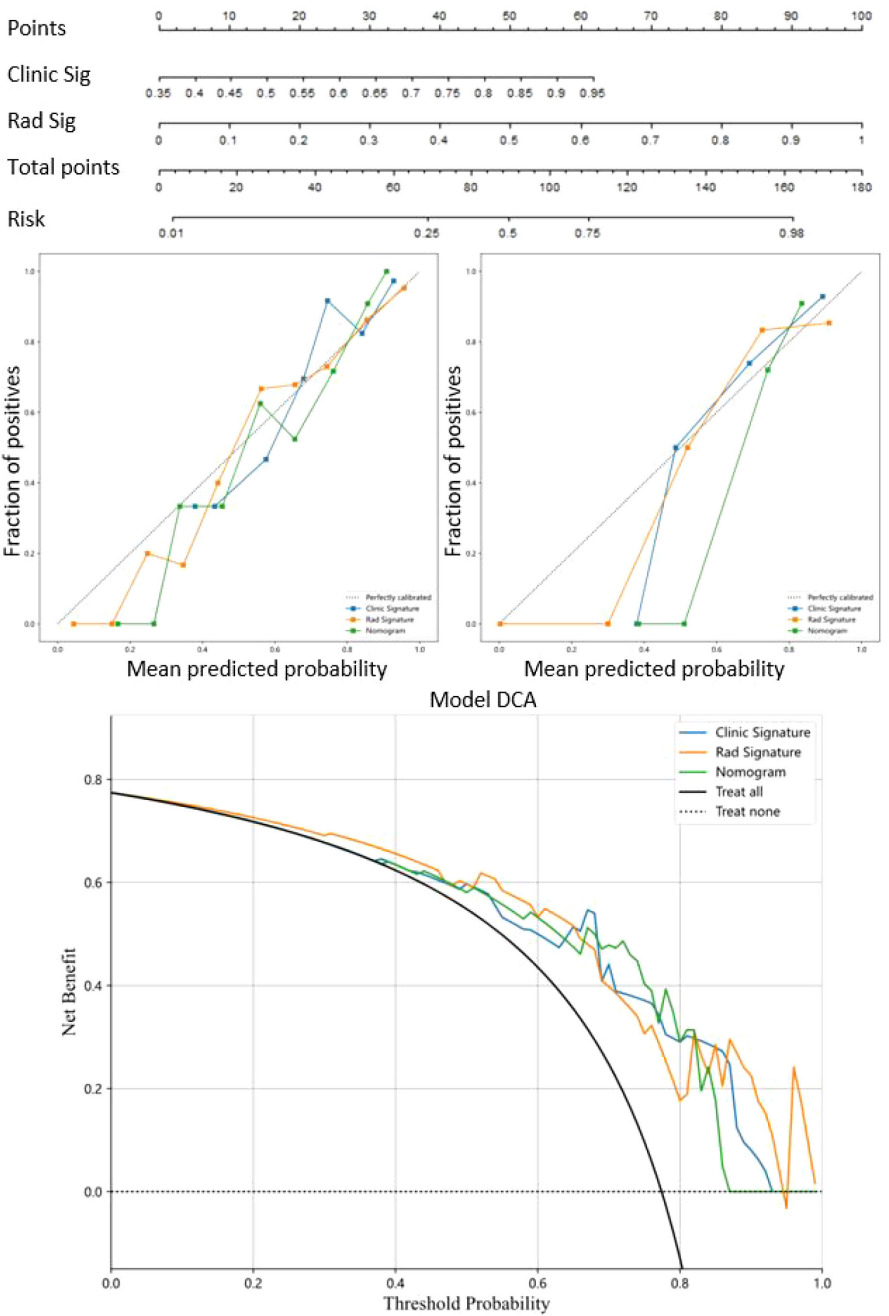
Nomogram model for prediction of Napsin A expression and evaluation of predictive utility. Nomogram model combined the radiomics signature models and clinical signature models. Calibration curves were used to evaluate the consistency between the predicted outcomes and the actual observations of Napsin A expression in training cohort and validation cohort. Decision curves analysis for the prediction of Napsin A expression in lung adenocarcinoma. The horizontal axis represents the threshold probability, and the vertical axis represents the net benefit rate corresponding to the threshold probability.

The nomogram visually represented the contribution of each predictor to Napsin A expression probability (**Figure 6**), facilitating individualized patient assessment. Calibration curves demonstrated strong concordance between predicted and observed Napsin A expression, with Hosmer-Lemeshow test P-values of 0.148 (training cohort) and 0.398 (validation cohort) (**Figures 6**). Decision curve analysis (DCA) indicated that the nomogram model offered greater net benefit across a broader range of threshold probabilities than standalone radiomic or clinical models (**Figure 6**), confirming its superior clinical utility.

## Discussion

Napsin A serves as a critical biomarker for differentiating primary lung adenocarcinoma from metastatic lesions, with its expression levels closely linked to tumor differentiation and prognosis. However, traditional immunohistochemical assessment requires invasive biopsy, which carries procedural risks (e.g., pneumothorax, bleeding) and may miss tumor heterogeneity due to limited sampling (22). Radiomics, by extracting quantitative features from CT images, offers a non-invasive alternative to characterize tumor biology comprehensively. This study aimed to bridge this gap by developing a nomogram integrating radiomic and clinical variables to predict Napsin A expression, addressing the unmet need for non-invasive molecular phenotyping in lung adenocarcinoma.

Our finding that female sex and non-smoking status correlate with higher Napsin A positivity (P<0.05) mirrors epidemiological studies linking these factors to adenocarcinoma histology. For example, Koezuka et al. (2022) demonstrated that non-smoking female patients exhibit a higher prevalence of lepidic-predominant adenocarcinoma, a well-differentiated subtype strongly associated with Napsin A expression. Similarly, Ren et al. (2022) reported that female gender correlates with TTF-1/Napsin A co-expression in adenocarcinoma, supporting the role of sex-specific biological pathways in alveolar epithelial differentiation (23). These similarities validate the clinical variables included in our model, as they reflect fundamental drivers of Napsin A expression.

The association between Napsin A negativity and spiculation sign/pleural indentation (P<0.05) aligns with Park et al. (2016), who linked these features to aggressive tumor phenotypes and reduced differentiation in EGFR-mutant adenocarcinomas (24). Conversely, the vacuolar sign—a marker of lepidic growth—was more prevalent in Napsin A-positive tumors, consistent with its association with well-differentiated subtypes in pure ground-glass nodules. These findings reinforce the radiomic model’s ability to capture microstructural characteristics indicative of Napsin A expression, bridging imaging phenotypes with molecular status.

Notably, most prior radiomics studies focus on predicting gene mutations (e.g., EGFR, ALK) rather than protein expression (21). This distinction may explain differences in feature importance; for instance, our model prioritizes texture features related to tumor-stroma interaction (e.g., GLCM cluster shade), whereas mutation-prediction models often emphasize vascular or metabolic parameters. Additionally, our single-center design with standardized CT protocols may yield more homogeneous radiomic features compared to multi-center studies, potentially affecting feature reproducibility (25).

The alignment between our clinical/radiographic findings and prior literature underscores the nomogram’s biological plausibility. However, differences in contrast administration protocols (e.g., fixed-dose vs. weight-based dosing) across institutions and reliance on manual segmentation in our study may limit direct comparability. Future multi-center studies with automated feature extraction are needed to validate these associations across diverse populations and imaging platforms.

The observed associations between radiographic features and Napsin A expression reinforce biological plausibility. Spiculation and pleural indentation, markers of fibrotic stromal response, were more prevalent in Napsin A-negative tumors, consistent with their role in tumor invasiveness and dedifferentiation. Conversely, the vacuolar sign, indicative of lepidic growth, was enriched in Napsin A-positive cases, supporting its link to well-differentiated subtypes. These findings extend prior radiomics research on tumor phenotyping, demonstrating its potential to non-invasively reflect molecular status.

The nomogram’s calibration (Hosmer-Lemeshow P > 0.05) and decision curve analysis (DCA) validate its clinical utility across threshold probabilities, surpassing traditional models. This aligns with advances in radiomic prediction of EGFR mutations and underscores its potential to reduce biopsy reliance, particularly in early-stage patients where non-invasive diagnosis is critical (26).

## Clinical implications and limitations

With balanced accuracy exceeding 80% in both cohorts, the nomogram could optimize preoperative workflows by triaging patients for biopsy. For example, those with high predicted Napsin A positivity might avoid invasive procedures, while low-prediction cases could prioritize tissue sampling to confirm molecular status (22). However, single-center data and a small validation cohort (n=62) limit generalizability, necessitating multi-center validation across diverse imaging platforms (27). Manual ROI segmentation, Although verified by ICC (≥0.75), remains a potential source of variability, warranting future adoption of automated algorithms (28).

While our study presents promising findings, several limitations warrant consideration. First, the sample size, particularly in the validation cohort (n=62), was relatively small. Second, radiomic feature reproducibility can be influenced by scanning parameters and segmentation methods, necessitating standardized protocols. Third, we acknowledge the challenge of potential overfitting, given that our model included 27 features while having only 57 Napsin A-negative patients in the training cohort. Future studies should consider more rigorous feature selection approaches to further reduce dimensionality while maintaining predictive performance. Fourth, we did not explore the combined predictive value of Napsin A with other molecular markers, such as EGFR mutations. Finally, all images were acquired at a single center using a single scanner, which may limit the model’s generalizability across different institutions and imaging platforms.

## Data Availability

The original contributions presented in the study are included in the article/supplementary material, further inquiries can be directed to the corresponding author/s.
